# The Hardness of Drinking Water Negatively while Socio-Economic Deprivation Positively Correlate with the Age-Adjusted Mortality Rates due to Cardiovascular Diseases in Hungarian Wine Regions

**DOI:** 10.3390/ijerph16183437

**Published:** 2019-09-16

**Authors:** János Nagy, Sándor Sipka, Sándor Sipka, Judit Kocsis, Zsolt Horváth

**Affiliations:** 1 Department of Nutrition, Faculty of Medicine, University of Debrecen, 4032 Debrecen, Hungary; 2 Institute of Cardiology, Faculty of Medicine, University of Debrecen, 4032 Debrecen, Hungary; 3 Bács-KisKun County Hospital, Centre of Onco-radiology, 6000 Kecskemét, Hungary

**Keywords:** age-adjusted death rate, cardiovascular diseases, index of deprivation, red wine, water hardness, white wine

## Abstract

We compared the age-adjusted death rates (AADR) for cardiovascular diseases (CVD) among 206,159 inhabitants analyzed between 2000 and 2010 in four wine territories of Hungary: Tokaj (white wines), Eger (mostly red wines), Balaton (mostly white wines), Szekszárd-Villány (mostly red wines) and Hódmezővásárhely (HMV) (not a wine region). The mortality rates were also assessed from the aspects of total hardness of drinking water and index of socio-economic deprivation (ID). We found the highest cardiovascular mortality in the Tokaj region and HMV. On the other hand, lower numbers of CVD were observed in Szekszárd-Villány, Balaton and Eger. These findings on cardiovascular mortality correlated negatively and significantly with the values of total hardness of drinking waters, which were low in Tokaj and HMV. They were higher in Szekszárd-Villány, Balaton and Eger. Additionally, and surprisingly, the mortality of CVD correlated positively and significantly with the ID values despite of the small numeric differences. The hardness of drinking water and the level of socio-economic state seem to have a greater impact on the mortality rate of CVD than the consumption of “red” or “white” dominant types of wines at a region. This study shows data on a population larger than 200,000 persons.

## 1. Introduction

Cardiovascular diseases (CVD) represent the leading cause of death in the countries of European Union. The least rates of mortality were found in Spain, France, Italy and Malta, as well as in Luxemburg and Sweden [1]. In this growing order, two tendencies are reflected: (1) The very advantageous position of Mediterranean countries, Spain, France, Italy and Malta. In these countries the inhabitants are known to eat the traditional “Mediterranean diet” rich of vegetables, fruits completed with drinking of red wines; (2) The high level of living standard, social development existing in Luxemburg and Sweden also can be protective factors against CVD. Inversely, the spatial association between social deprivation and CVD mortality was observed in USA [2]. Regarding CVD the protective effects of red wines rich in anti-oxidants was suggested [3]. Furthermore, concerning the positive cardio-protective effects of “Mediterranean diet”, the role of red wines and slight alcohol consumption were found to be more important than that of vegetables, fruits, fish and dairy products [4]. Thus, it can be concluded that up to now a much greater attention has been paid toward the “red” than the “white” characters of wines from the aspect of public health.

In the last decades, the positive role of hard drinking water rich in magnesium and calcium has been observed in the prevention of CVD [5]. “Hard water consumption seems to be protective against CVD” was documented as the main conclusion of the latest systemic review with meta-analysis of case-control studies based on 643 articles, ending with the following remark: “However, even further data on larger populations are required to confirm these results” [6]. It was important that two new studies were published from Slovakia confirming the previous positive statement on the protective role of hard water against CVD [7,8].

In the current work, we investigated the potential roles of “red” and “white” wine consumptions, as well as the hardness of drinking water and the socio-economic state on the CVD mortality in four historic wine regions of Hungary and in a “control” city (without great wine tradition) tested in a large population over 200,000 persons. In the wine regions (2-2 with dominantly red and white wines), the age-adjusted death rates (AADR) were calculated for 206159 subjects counted for 100,000 persons who died of CVD between 2000 and 2010 during 11 years. [9].

### The Dominant Types of Wines and the Settlements of Territories

The wine regions and the types of dominant wines are as follows: Tokaj—special, partly *Botrytis cinerea* related local types of “white” wines, named as “Tokaj wines”, Eger—mostly commercial “red” wines, Balaton—mostly commercial “white” wines, Szekszárd-Villány—mostly commercial “red” wines, Hódmezővásárhely (HMV)—“control” territory, not a wine region. The data derive from the following 12 settlements: Tokaj region: Tokaj, Sárospatak, Sátoraljaújhely; Eger region: Eger; Balaton region: Badacsonytomaj, Badacsonytördemic, Balatonfüred, Dörgicse, Csopak; Szekszárd-Villány region: Szekszárd, and Villány and as a “control” Hódmezővásárhely.

## 2. Methods

### 2.1. Age-Adjusted Death Rate

The data of persons who died in Hungary during the studied 11 years long period of 2000–2010 were provided by the Central Bureau of Public and Electronic Administration and the Hungarian Central Statistical Office (HCSO). The data were grouped according to settlements, sex, age and diagnosis. The CVD diagnoses were set up according to the International Statistical Classification of Diseases and Related Health Problems (ICD). We calculated the age-adjusted death rates (AADR values) for 100,000 subjects in every region focusing on the whole period, and not on the annual changes or differences.

“AADR value” = total number of dead persons due to CVD/100,000 inhabitants/11 years.

### 2.2. The Measurement of the Indicators of Hardness of Drinking Water

The data containing hundreds of items on the main indicators of hardness of drinking water: calcium (mg/L), magnesium (mg/L) and total hardness (CaO mg/L) were provided and controlled by the experts of National Institute of Health (Budapest) based on their measurements carried out officially and regularly from at least 50-50 points of the 12 settlements in the five regions. However, there were not data available from every year and every point of measurements. Otherwise, the results did not show almost any remarkable changes between 2000 and 2010. Therefore, we used only the summarized averages of Ca (mg/L), Mg (mg/L) and total hardness (CaO mg/L) for each region.

### 2.3. Calculation of the Index of Socio-Economic Deprivation (ID)

The comparison of the socio-economic states of five regions occurred by the calculation of the index of deprivation (ID) according to Juhász and coworkers [10] using the data of HCSO gained by the latest national census in 2011. Deprivation means retardation, underdevelopment compared to the national average. The negative sign means a better social state. It was important in the choice of regions that we tested only their comparable urban populations representing living standards higher than the average of Hungary. They all showed negative ID values with small numeric differences. The values of ID were calculated from data of HCSO using the following indicators: (1) annual income/ person in the region; (2) ratio of persons in the population under 15 years not finishing the elementary school; (3) ratio of joblessness in the population of 15–74 years; (4) ratio of families with one parent; (5) proportion of families with three or more children; (6) number of persons/rooms; (7) the number of cars/100 persons. Additionally, the level of health care service, medical background, medicine consumption, the basic conditions for diagnosis and treatment of CVD were similar (better than the national average) among these regions and people tested. Otherwise, the level of medical service is partly reflected in the values of ID. They could reflect the social states which did not change too much during 11 years.

### 2.4. Statistical Analysis

The AADR values calculated annually during the 11 years were summarized then adjusted to the population sizes of the five different regions [9]. Pearson’s correlations were calculated between (a) AADR of CVD and hardness of drinking water; (b) AADR of CVD and indices of socio-economic deprivation (ID). The values of total hardness of drinking water were expressed using average and standard deviation (SD) from at least 50-50 points of measurements carried out by the National Institutes of Health (Budapest) regularly (but not every year) in each settlement. *p* < 0.05 values were regarded to be significant. The IBM SPSS ver. 24 program (IBM Corp, Armonk, NY, USA) was used for the calculations.

## 3. Results

### 3.1. The Number of Population, Age-Adjusted Death Rates Due to Cardiovascular Diseases, the Hardness of Drinking Water and Index of Deprivation in the Five Regions Between 2000 and 2010

Table 1 shows the summarized data of the number of populations, the averages of age adjusted death rates (AADR) due to cardiovascular diseases, the hardness of drinking water and the indices of deprivation (ID) in the five regions. The highest values of CVD mortality (AADR: 5955) were observed in the Tokaj “white wine” region and in the not wine region HMV (AADR: 5178). On the other hand, in Szekszárd-Villány (AADR: 3907) and Eger (AADR: 4191) in these “red wine” regions, the values were lower than in the previous regions, suggesting the confirmation of the earlier international observations on the potential protective role of red wines [3,4]. However, it became also apparent that in the case of “white wine region Balaton”, both the highest level of living standard (least value of ID: −1.22) and the high value of hardness of drinking water (249.20 CaO mg/L) could contribute to the decrease in CVD mortality compared to Tokaj and HMV.

### 3.2. The Indicators of Hardness of Drinking Water in the Five Territories

Table 2 shows the average + SD values of the indicators of hardness of drinking water: magnesium (mg/L), calcium (mg/L) and total hardness (CaO mg/L). The growing order is in the values of total hardness (CaO mg/L) as follows: HMV (81.90), Tokaj (138.60), Eger (194.90), Balaton (249.20), Szekszárd/Villány (294.20). The Hungarian classification of grades (mg/l) in the total hardness of drinking waters are as follows: soft: 0–140 (HMV and Tokaj), medium hard: 140–210 (Eger), hard: >210 (Balaton and Szekszárd/Villány).

### 3.3. A Negative Correlation between the AADR Values Due to Cardiovascular Diseases and Total Hardness of Drinking Water in the Five Regions in a Population Larger than 200,000 Persons

**Figure 1:**
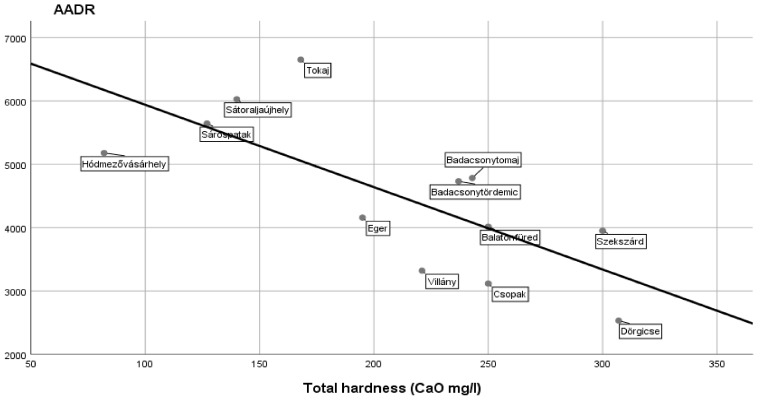
A negative correlation (r = −0.731; *p* < 0.007) between the age-adjusted death rates (AADR) values due to cardiovascular diseases and total hardness of drinking water found in the settlements of five regions in a population larger than 200,000 people.

A statistically significant negative correlation was found between the AADR values of CVD and the hardness of drinking waters: r = −0.731, *p* < 0.007. The results derived from 12 settlements of the five regions are demonstrated in Figure 1. It is of note that this study was carried out on a population with higher number than 200,000 persons.

### 3.4. A Positive Correlation between the AADR Values Due to Cardiovascular Diseases and Indices of Socio-Economic Deprivation in the Five Regions in a Population Larger than 200,000 Persons

**Figure 2:**
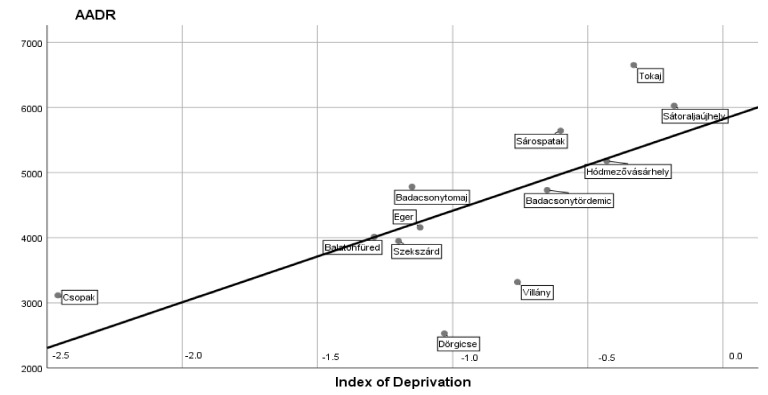
A positive correlation (r = 0, 690; *p* < 0.013) between the AADR values due cardiovascular diseases and indices of socio-economic deprivation found in 12 settlements of the five regions in a population larger than 200,000 persons.

A statistically significant positive correlation was found between the AADR values due to cardiovascular diseases and indices of socio-economic deprivation r = 0.690, *p* < 0.013. The results derived from 12 settlements of the five regions are demonstrated in Figure 2. It is important to stress that this strong linkage could be verified despite of the small numeric differences in the ID values. To find this significant correlation it was advantageous that the calculation was carried out on a population larger than 200,000 persons.

### 3.5. The AADR Values Due to Cardiovascular Diseases Presented from the Aspect of “Red” or “White” Dominant Types of Wines in the Regions

**Figure 3:**
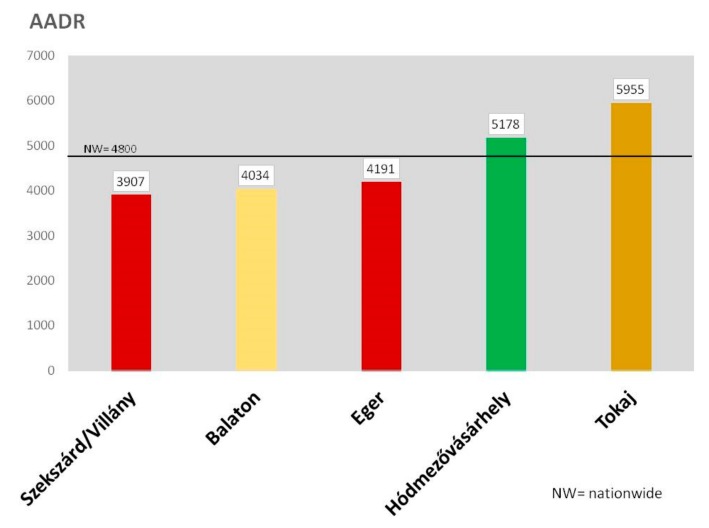
The AADR values due to cardiovascular diseases presented from the aspect of “red” or “white” dominant types of wines in the regions.

Figure 3 demonstrates that compared to the nationwide AADR result (4800) of CVD both HMV (5178) and Tokaj (5955) show higher, whereas Szekszárd/Villány (3907), Balaton (4034) and Eger (4191) lower values. It can be seen that the relatively good protective results of the latter three regions cannot be explained by the “red” or” white” types of the dominating local wines, because both types occur among them: Szekszárd/Villány and Eger with “red”, Balaton with “white” wines.

## 4. Discussion

The main results of our study are as follows: (a) a significantly negative correlation is shown between the hardness of drinking waters and the AARD values of CVD in the five regions; (b) a significantly positive correlation is found between the AADR values of CVD and the indices of socio-economic deprivation; (c) both results are based on data which derive from a population larger than 200,000 persons. (d) The hardness of drinking water and the level of socio-economic state seem to have a greater impact on the mortality rate (AADR) of CVD than the consumption of “red” or “white” dominant types of wines at a region.

Our starting intention was to study the correlations between AADR of CVD at the various regions from the aspect of consumption of “red” or “white” dominant types of wines. The regions were chosen concerning this idea: two-two historic wine regions from both types and a “control” one without international traditions in wine making. We also tried to study territories without great socio-economic differences. Therefore, the data were collected from cities, where the living standards were higher than the average of Hungary, despite of the small numeric differences in their negative ID values. We realized the importance of the hardness of drinking water only in a later phase of study. Additionally, it became our surprise that how important were to pay attention even on the slight differences in the ID values. Finally, we recognized that the differences found in the cardiac mortality rates could be well explained by the data of hardness of drinking waters and the values of ID in parallel, however, by the data of wine types, not. The statistical differentiation between the roles of hard water or social deprivation is very difficult. Eventually the greater impact of hard water can be suggested by the faint argument that the numeric value of its correlation co-efficient is 0.731 versus 0.680.

It is of note, that these findings are valid only for a defined period of time and population living in these five territories of Hungary. Since the time when these data were collected, the circumstances have changed a lot. However, the period of the analyzed 11 years was a very fortunate choice to study the potential effects of wines. Majority of the people who died between 2000 and 2010 were born between 1930 and 1950, and belonged to a generation which already lived in small cities but was still engaged very strongly with their relatives living in villages. Their wine consumption was traditionally and continuously tied to their own production at home or to the small family cellars according to the “goulash cooking” fashion. The “amount/person/year value” of pure ethyl-alcohol consumption was also similar in the four wine territories [11].

Nevertheless, these people mainly drank the piped drinking waters of their settlements during their whole life. Additionally, they were mainly wine and not beer drinkers. They did not know yet the newly dominating bottled commercial/mineral waters and various soft drinks. Since that the consumption of beer also increased in the whole country.

Of course, the hardness of various region-specific wines needs also an attention. According to a detailed chemical analysis, a slightly less concentration of calcium and magnesium was found in the wines of Tokaj than in those of Eger, Balaton and Szekszárd-Villány, independently of the red or white characters. (May be that these results reflect the hardness of drinking waters at the region?) However, the technology could modify the trace element contents of these wines [12].

Anyhow, these results clearly show that the data on AADR of CVD have significant relations only with the values of hardness of drinking waters and the socio-economic deprivation. Yet, we do not exclude that red wines of Szekszárd-Villány and Eger may have uncertain protective effects on CVD, as our results coincided with some earlier similar observations [3,4]. Concerning the high value of AADR of CVD in the Tokaj region, however, it is important to declare that the soft grade of drinking waters and the worst value of ID could be the main causative factors, and not the chemical character of these wines. In the “control” city, Hódmezővásárhely, similarly to the Tokaj, also the soft grade of drinking water and the relatively bad value of ID could result in the high rate of CVD mortality. In the good results from the Balaton region, both the high values of water hardness and living standard could play the dominating roles. Thus, the consumption of “red” or “white” types of wines was not a significant factor to influence the cardiologic mortality rates in the traditional Hungarian wine regions.

In the explanation of mechanisms how social background and the hardness of drinking water act on CVD, it is out of question that people living in higher living standards may rather afford such life styles which result in a more effective CVD prevention than poverty. Concerning hard drinking water, however, it is of note that it may have a protective role against the early stages of atherosclerosis [13] and helps to preserve elastic arteries [8].

## 5. Conclusions

This study showed and confirmed the existence of a significantly negative correlation between the mortality rates of CVD and the hardness of drinking waters using data of a population larger than 200,000 persons [5], furthermore, the existence of a significant positive correlation between the mortality rates of CVD and socio-economic deprivation [2]. This investigation which was carried out on the population of four historical wine regions of Hungary and on a “control” city suggests that the “red” or “white” types of dominating consumed wines do not have such an importance in the development of cardiac diseases as the hardness of drinking water and the social-economic deprivation at a region. Additionally, these findings are drawing attention to the awareness in the liquid (water) drinking habits, preferring to drink hard waters [14]. “Some bottled water may be salubrious” [15,16]. Nevertheless, these results demonstrate statistical facts concerning the roles of “red” and “white” wines in CVD mortality helping to avoid any positive or negative preconceptions. Therefore, an “alternating, intermittent and moderated” consumption of all these wines independently of their “red” or ”white” types, may remain further an important element of gastronomic and general human culture, but not as potent antidotes of CVD.

## Figures and Tables

**Table 1:** The number of populations, age-adjusted death rates due to cardiovascular diseases, the hardness of drinking water and index of deprivation in the five regions between 2000 and 2010.

Parameters	Tokaj	Eger	Balaton	Szekszárd/Villány	*Hódmezővásárhely*
Population	33,917	56,981	30,833	37,268	47,160
Cardiovascular mortality (AADR)	5955	4191	4034	3907	5178
Drinking water total hardness (CaO mg/L)	138.60	194.90	249.20	294.20	81.90
Index of deprivation	−0.36	−1.1	−1.22	−1.17	−0.43

AADR—Age-Adjusted Death Rate.

**Table 2:** The indicators of hardness of drinking water in the five territories.

Element/Compound	Unit	Tokaj	Eger	Balaton	Szekszárd/Villány	*Hódmező-Vásárhely*
Magnesium	mg/L	24.50	26.80	46.20	39.70	14.50
Average ± SD		± 4.4	± 5.3	± 5.1	± 6.2	± 7.7
Calcium	mg/L	2.40	86.20	115.40	8.20	27.60
Average ± SD		± 0.35	± 1.13	± 17.6	± 7.8	± 15.1
Total hardness	CaO mg/L	138.60	194.90	249.20	294.20	81.90
Average ± SD		± 31.3	± 30.8	± 33.7	± 40.6	± 37.3

SD Standard Deviation.
